# Climate change not to blame for late Quaternary megafauna extinctions in Australia

**DOI:** 10.1038/ncomms10511

**Published:** 2016-01-29

**Authors:** Frédérik Saltré, Marta Rodríguez-Rey, Barry W. Brook, Christopher N Johnson, Chris S. M. Turney, John Alroy, Alan Cooper, Nicholas Beeton, Michael I. Bird, Damien A. Fordham, Richard Gillespie, Salvador Herrando-Pérez, Zenobia Jacobs, Gifford H. Miller, David Nogués-Bravo, Gavin J. Prideaux, Richard G. Roberts, Corey J. A. Bradshaw

**Affiliations:** 1 The Environment Institute and School of Biological Sciences, The University of Adelaide, North Terrace, Adelaide, South Australia 5005, Australia; 2 School of Biological Sciences, Private Bag 55, University of Tasmania, Hobart, Tasmania 7001, Australia; 3 School of Biological, Earth and Environmental Sciences, University of NSW, Sydney, New South Wales 2052, Australia; 4 Department of Biological Sciences, Macquarie University, Sydney, New South Wales 2109, Australia; 5 Australian Centre for Ancient DNA, The University of Adelaide, Adelaide, South Australia 5005, Australia; 6 Centre for Tropical Environmental and Sustainability Studies, James Cook University, Cairns, Queensland 4878, Australia; 7 Centre for Archaeological Science, School of Earth and Environmental Sciences, University of Wollongong, Wollongong, New South Wales 2522, Australia; 8 Department of Archaeology and Natural History, School of Culture, History and Language, Australian National University, Canberra, Australian Capital Territory 0200, Australia; 9 Department of Biogeography and Global Change, National Museum of Natural Sciences—Spanish Research Council (CSIC), c/José Gutiérrez Abascal 2, 28006 Madrid, Spain; 10 Institute of Arctic and Alpine Research, Geological Sciences, University of Colorado, Boulder, Colorado 80309-0450, USA; 11 Environment and Agriculture Curtin University Perth, Perth, Western Australia 6102, Australia; 12 Center for Macroecology, Evolution and Climate, Natural History Museum of Denmark, University of Copenhagen, Copenhagen DK-2100, Denmark; 13 School of Biological Sciences, Flinders University, Bedford Park, South Austalia 5042, Australia

## Abstract

Late Quaternary megafauna extinctions impoverished mammalian diversity worldwide. The causes of these extinctions in Australia are most controversial but essential to resolve, because this continent-wide event presaged similar losses that occurred thousands of years later on other continents. Here we apply a rigorous metadata analysis and new ensemble-hindcasting approach to 659 Australian megafauna fossil ages. When coupled with analysis of several high-resolution climate records, we show that megafaunal extinctions were broadly synchronous among genera and independent of climate aridity and variability in Australia over the last 120,000 years. Our results reject climate change as the primary driver of megafauna extinctions in the world's most controversial context, and instead estimate that the megafauna disappeared Australia-wide ∼13,500 years after human arrival, with shorter periods of coexistence in some regions. This is the first comprehensive approach to incorporate uncertainty in fossil ages, extinction timing and climatology, to quantify mechanisms of prehistorical extinctions.

## Abstract (web summary)

Global megafaunal extinctions took place in the late Quaternary, yet the relative impact of climate and humans in the faunal collapse is unclear. Here, the authors show that megafaunal extinctions in Australia were independent of climate variability and took place approximately 13,500 years after human arrival.

## Graphical abstract

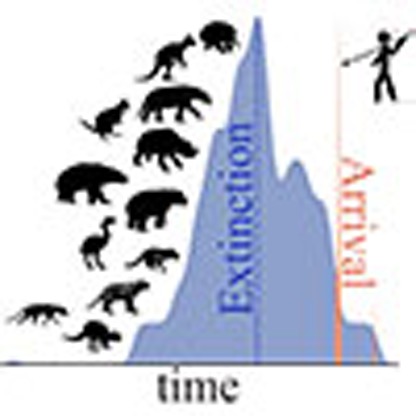

The late Quaternary was a period of rapid and widespread extinction of about 65% of ‘megafauna' genera (that is, large vertebrates with mature individuals >40 kg)^1^. Although climatic shifts probably caused some of the largest mass extinctions earlier in Earth's history^2^, the role of climate in Quaternary faunal collapse is still hotly debated^1^,^3^,^4^,^5^, because many of these extinctions coincided with human colonization^6^. The initial arrival of humans in Australia and New Guinea (then connected by a land bridge and hereafter referred to collectively as ‘Sahul'), and extinction of the megafauna took place close to, or beyond, the limit of radiocarbon (^14^C) dating (55–45 kyr ago, kyr=10^3^ years), leading to uncertainty whether humans had a specific and important role in driving continent-wide extinctions of megafauna in Sahul^1^. The ‘climate-driven' hypothesis of Australian extinction states that changes in climate variability coupled with severe aridification from about the last 430 kyr caused the extinctions^7^, despite evidence that climate was relatively stable during the period of most disappearances^8^,^9^. Alternatively, the ‘human-driven' hypothesis stipulates that hunting was the major cause of extinctions^8^,^10^, and/or that humans modified natural fire regimes enough to alter vegetation communities and disrupt trophic dynamics^10^. A third hypothesis emphasizes a possible human–climate synergy, in which human impacts delivered the *coup de grâce* to populations already compromised by climate-driven environmental changes^1^,^5^.

Limits to ^14^C dating have made it difficult to construct an accurate chronology of species losses relative to major climatic or archaeological transitions^3^. The primary data describing the timing of megafauna extinctions are from estimated ages of fossilized remains^11^,^12^,^13^,^14^,^15^, but the final time of extinction of any long-disappeared population inevitably diverges from the youngest records due to a bias introduced by incomplete sampling or taphonomy (the Signor–Lipps effect)^16^. Many inferential methods have been developed to estimate the time gap between the last dated fossil and the final extinction date, but their efficiency varies with the type of extinction and sampling density over time^17^. In addition to these taphonomic and statistical issues, the Australian fossil data set has traditionally been of questionable (that is, sparse and variable) quality^14^,^18^,^19^, until the recent development of a general tool for assessing the reliability of age estimates for fossils^20^.

Here we use the largest, most diverse and rigorously quality-controlled data set in existence for Sahul, to investigate the role of variation in climate on megafauna extinction in Sahul and to estimate the window of coexistence of megafauna and humans, indicating the likelihood of a human-driven alternative (i.e., we hypothesize that the longer the overlap, the more time humans would have had to alter the environment and affect megafauna). We achieve this by (i) developing a robust chronological framework that reduces uncertainties in the timing of megafauna extinctions and first human occurrence; and (ii) testing the correlation of the chronology of megafauna extinctions (i.e., the number of genera that went extinct through time) against six high-resolution climate records and hindcasts. We show that (i) over the last 120 kyr, the chronology of megafauna extinctions is not correlated with changes in climate variability or more arid events at a continental scale; and (ii) the period of human–megafauna coexistence lasted ∼13.5 kyr across the continent as a whole, but with shorter periods of overlap probably in any particular region. These findings imply that human pressure, rather than climate change, was most likely to be the main driver of these extinctions.

## Results

**Figure 1:**
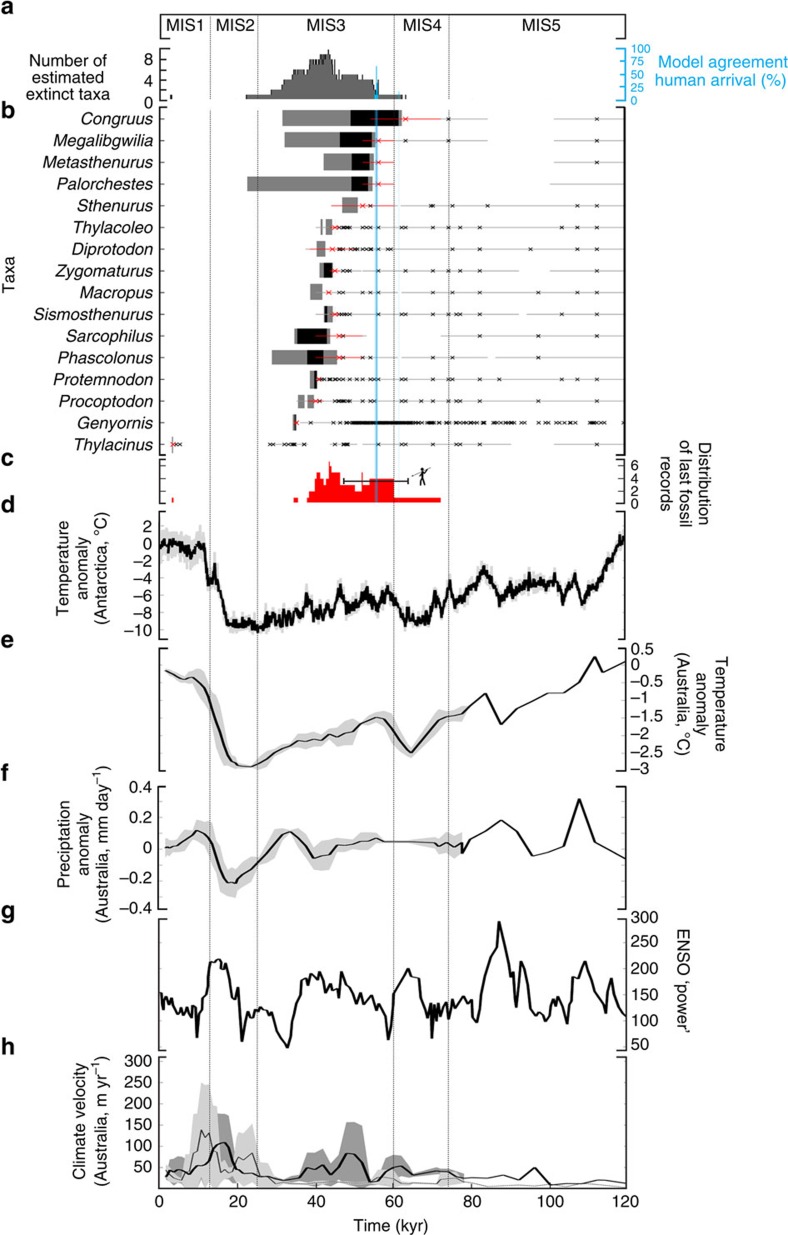
Period of human–megafauna overlap and climate variation in Sahul. (**a**) Distribution of extinction times for all megafauna genera with a model agreement >50% (grey barplot, left *y*-axis) and distribution of the percentage of model agreement to infer the time of first human occurrence (blue barplot, right *y*-axis). Marine isotope stages (MISs) 1 to 5 (ref. 26) are shown across the top axis for temporal reference. (**b**) Percentage agreement between six models^17^ used to infer both the time of extinction for each megafauna taxon (black=100% model agreement, grey=50% and white=0% agreement) and the time of first human occurence (from dark blue=100% agreement to white=0% agreement) from fossil and archaeological records. For each taxon, crosses and grey lines denote fossil ages and their 2*σ* uncertainties; the red crosses and lines show the most recent fossil ages and their 2*σ* uncertainties. The cutoff at 50% is the maximum threshold to compute an extinction window for each taxon. (**c**) Frequency distribution of the youngest age for each genus of all 16 genera (red plot), accounting for dating uncertainties, and temporal range of first human occurrence (black line) established from ages±2*σ* uncertainties of the oldest archaeological evidence rated as high quality. (**d**) Mean annual temperature anomalies relative to the present day (±s.d., grey-shaded envelop), calculated from the Antarctica EPICA Dome C ice core^22^ and corrected for time-resolution sampling bias. (**e**) Mean annual temperature and (**f**) precipitation anomalies relative to the present day in Sahul, calculated from HadCM3 palaeoclimate simulations^47^. (**g**) Variation in ENSOp (dimensionless) estimated from the Zebiak–Cane coupled ocean–atmosphere model forced only by changing orbital parameters^23^. (**h**) Velocity of climate change^34^ calculated from mean annual temperature (dark grey) and precipitation (light grey) in **e** and **f**, respectively. In **e**, **f** and **h**, the time resolution of each variable is standardized using a 4 kyr running mean starting 80 kyr ago; the bold lines indicate the median value, whereas the lower and upper limits of their grey-shaded envelopes are determined by the first and third quantiles, respectively.

To estimate an accurate chronology for the extinct megafauna and earliest human arrival in Sahul, we developed an ensemble-hindcasting model framework. We applied a set of six frequentist statistical methods (see Supplementary Methods and Supplementary Table 1) to high-quality rated^20^ time series for 16 megafauna genera and to the archaeology of first human colonization of Sahul (Fig. 1 and Table 1); these represent all of the available data (for both the megafauna and the archaeology time series) suitable for analysis (see details on the quality-rating approach in Methods). We developed a multi-model comparison (see Methods) to estimate the temporal window of coexistence between humans and megafauna. Our results show a peak in extinction events at 42.1 kyr (that is, median value of the distribution of estimated extinction dates in Fig. 1a) with a continent-wide confidence interval (CI_megafauna_) ranging from 36.7 to 48.1 kyr (75th and 25th percentiles of the distribution of estimated extinction dates in Fig. 1a, respectively).

The earliest estimated extinctions for *Congruus*, *Megalibgwilia*, *Metasthenurus*, *Palorchestes* and *Sthenurus* were between 61 and 51 kyr, followed by 10 genera (all remaining genera, excluding *Thylacinus*) estimated to have been driven extinct between 44 and 35 kyr. We provide similar statistical support for the first human arrival in Sahul at 55.6 kyr (CI_human_=55.4–55.7 kyr for the 75th and 25th percentiles, respectively), which is similar to other recent estimates^11^,^21^ and indicates a median window of continent-wide coexistence of 13.5 kyr (CI=7.3–19 kyr, calculated as lower CI_human_−upper CI_megafauna_ and upper CI_human_−lower CI_megafauna_) before the peak of megafauna extinction (Fig. 1b). Interpreting the youngest dated fossil records for each taxon as the actual time of extinction (ignoring the Signor–Lipps effect) would predict an older extinction peak at 50.8 kyr (CI_megafauna_=45.9–57.1 kyr, Fig. 1c), whereas the oldest dated archaeological evidence for human arrival would be 55.5 kyr (CI_human_=47.3–63.7 kyr; Table 1).

To explore the potential role of variation in climate conditions on this extinction chronology, we (i) tested whether climate conditions during the estimated period of megafauna extinction are unique compared with previous climatic conditions over the preceding 60 kyr (i.e., from 120 to 60 kyr ago); and (ii) correlated the number of extinct taxa with climatic conditions from 120 kyr ago until the end of the extinction peak (about 35 kyr; Fig. 1a). We expressed climate variation over the last 120 kyr using several proxies (see Methods), including reconstructed temperature anomalies from central East Antarctica^22^, modelled El Niño/Southern Oscillation variability ‘power' (ENSOp)^23^, and anomalies and the velocity of changes in both hindcasted temperature and precipitation from Sahul (Fig. 1). Other climate proxies have previously been used to investigate the effects of climate shifts on megafauna extinctions^13^,^24^,^25^, but these potentially describe local climatic conditions rather than continent-wide trends.

The relatively arid conditions dominating marine isotope stage (MIS) 3 from 59 to 24 kyr^26^ (Fig. 1) are not unique. There were periods before MIS3 when climate was considerably more arid and yet these genera persisted. For example, mean annual temperatures in Antarctica were up to 4 °C warmer during the estimated period of megafauna extinctions than at the end of MIS4 (the interval from 72 to 59 kyr^26^), representing a shift of temperature anomaly in Antarctica (i.e., temperature differences from the present day) from a mean of −10 to −6 °C (Fig. 1d). Temperatures in Sahul indicate a similar trend, although MIS3 warming was <1 °C higher than MIS4 conditions (a shift from a mean anomaly of −2.5 °C at MIS4 to −1.5 °C at MIS3; Fig. 1e) and precipitation anomaly decreased by <1 mm per day during the MIS4/3 transition (Fig. 1f). These temporal trends in climate are supported by higher ENSOp during MIS3 (Fig. 1g), which indicates more arid conditions. Climate velocity proxies indicate that genera would have had to shift their ranges by moving up to 10 and 150 m per year (Fig. 1h), to track shifts in temperature and precipitation, respectively, during MIS3. During MIS5 (123–72 kyr)^26^, however, mean temperatures in Antarctica were about 2 °C warmer (*T* °C_MIS5_ anomaly=−3 °C versus *T* °C_MIS3_ anomaly=−5 °C; Fig. 1d) and both temperatures and precipitation in Sahul appear to have been higher than during MIS3 (up to 0.4 mm per day of precipitation and >1.5 °C warmer). Higher ENSOp at MIS5 (ENSOp_MIS5_=300 versus ENSOp_MIS3_=200; Fig. 1g) indicates more extreme aridity between 100 and 80 kyr than during MIS3.

**Figure 2:**
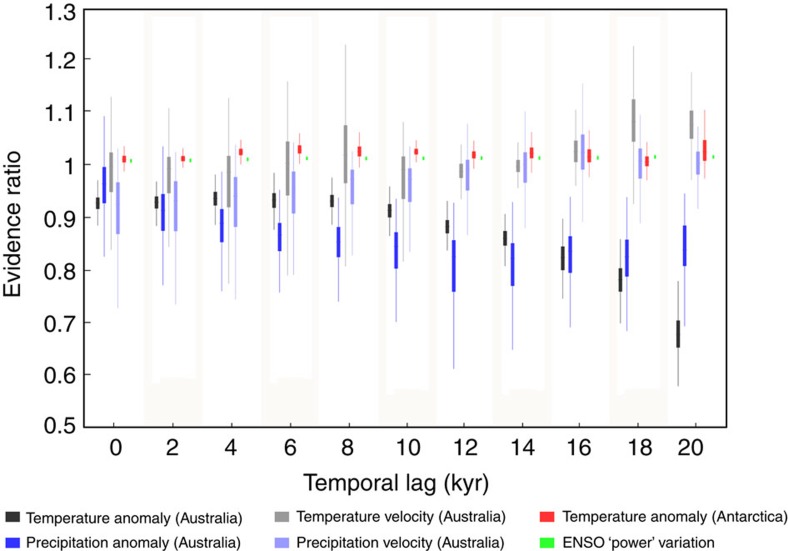
Boxplot of information-theoretic evidence ratios (ERs) comparing two regression models. Models are fitted on variation in the climate proxy against the number of extinct genera (estimated from model agreement outputs) from 120 to 35 kyr ago, as a function of temporal lag between climate variation and time of extinction. The first regression model assumes a linear relationship between climate variation and the number of extinct genera, whereas the second model assumes no such relationship (that is, no climate effects on megafauna extinction). An ER of >> 3 would indicate support for the slope (climate effect) model^27^; thus, the observed ratios of ≤1.2 provide no evidence of a climatic influence on extinction.

To test whether climate could have affected the number of genera that went extinct in Sahul, we used an information-theoretic evidence-ratio approach (evidence ratio (ER) of Akaike's information criterion (AIC) model weights; *ER*>>3 would indicate strong evidence of a climate effect; see Methods and ref. 27). We fitted two regression models to the number of extinct genera every 2 kyr (Fig. 1a) against the value of each of the six climate proxies at the same date (Fig. 1d–h). The first model assumes a linear relationship between climatic variation and the number of extinct genera, whereas the second assumes no relationship (intercept-only ‘null' model). The ER quantifies the extent of support for the linear model (that is, climate related to the number of extinct genera) relative to the ‘null' model (no climate effect)^27^. We also accounted for possible temporal lags, as extinctions could be driven by earlier climatic variation^8^,^25^ (see Methods). We detected no evidence of a correlation between the timing of extinction events and variation in climate based on any of the measures of climate used here. ERs were ≤1.2 for all climate indices whatever the temporal lag (Fig. 2), based on either the estimated model-agreement extinction outputs (Fig. 1a) or the distribution of last fossil ages for each taxon (Fig. 1c and Supplementary Fig. 4). This demonstrates no support for the climate-driven extinction hypothesis.

The lack of statistical support for a linear relationship between the number of extinct genera and any of the climate proxies (Fig. 2) indicates that the role of climate change was either negligible, secondary and/or synergistic with humans, or operated at spatial scales too restricted to be detected in the continent-wide megafauna data set. There are also physiological and ecological reasons to expect that any increase in aridity would have favoured large-bodied animals^15^. However, local environmental changes^13^,^24^,^25^ could have led to local extirpation of genera and habitat fragmentation, possibly exacerbating human predation on megafauna, as has been suggested for Eurasia^5^,^28^ and South America^29^. This continent-wide result is consistent with the fact that there are no unique extreme climate conditions during MIS3 that could plausibly lead to the inferred accumulation of extinction events during this period (Fig. 1a). Indeed, even more extreme conditions occurred during MIS5, when all the genera we tested were extant.

Furthermore, even under the fastest climate velocities in MIS3, megafauna taxa would have needed to migrate no more than 150 m per year (Fig. 1h), to track favourable climatic conditions. At such a low spatial velocity of climate change, variation in vegetation composition (i.e., expansion of open shrublands, grasslands and woodland) as observed in pollen records^30^,^31^,^32^ and carbon isotope signatures^10^,^33^ would have occurred slowly; thus, herbivore genera would have easily been able to migrate in response to shifts in the distribution of food and water resources^15^. In addition, climate velocity estimates are sensitive to the spatial resolution of the analysis (that is, velocity increases at coarser spatial resolutions)^34^,^35^; hence, we are likely to have overestimated the real velocities that the megafauna experienced *in situ*, owing to the 1 × 1° resolution of our climate data set.

## Discussion

Both the timing of megafauna extinction and first human arrival in Sahul have long been controversial given the lack of reliable data and bias introduced by the Signor–Lipps effect. Having now accounted for these deficiencies, our results support the general conclusions of previous studies^11^,^21^, but they are much better constrained by more diverse and expanded data sets that have been rigorously scrutinized using the best-available analytical tools. It is generally accepted that humans were present in Australia by 48 kyr^11^,^36^,^37^. Our modelled age estimate of 55.6 kyr reflects the early human colonization of northern Australia^21^,^38^, but geographic gaps in high-quality data (see Supplementary Fig. 1) prevent a reliable interpretation of the pattern of human dispersal across Sahul. It is clear, however, that humans were present in Tasmania by 39 kyr^36^ and in the arid centre of Australia by 35 kyr^39^.

The 13.5-kyr window of human–megafauna coexistence estimated is sufficiently long for groups of hunter–gatherers to disperse and become established across Sahul^40^ and to extirpate many megafauna species without a wave of rapid, continent-wide overkill. These results do not dismiss the possibility of rapid human overkill at more local/regional scales, nor that the longer the period of coexistence, the more time megafauna would have had to adapt to human presence and become more immune to their impacts. However, we currently lack direct evidence for human–megafauna interactions, possibly because of taphonomic loss and sampling bias^41^. Nonetheless, quantitative models have demonstrated that even small groups of hunter–gatherers living across a vast continent and using stone-based technologies could feasibly exterminate species with low population growth rates, such as large-bodied mammals^42^. This conclusion could not be supported by results based only on a direct reading of the fossil record, because it would be biased by the uncorrected Signor–Lipps effect^16^ (Fig. 1c). By including only reliably dated megafauna and archaeological records in our models, and explicitly correcting for the Signor–Lipps effect, the duration of human–megafauna coexistence can be estimated with much higher statistical confidence. Our approach allows us to discriminate between the various proposed extinction mechanisms^11^ for the late Quaternary megafauna of Sahul and has revealed that climate change was not a continental driver of extinction of these genera. The same approach could similarly provide new insights into the causes of late Quaternary megafauna extinction events in other regions of the world.

## Methods

### Megafauna extinction and first human occurrence

We used six established frequentist methods (see full model descriptions in Supplementary Methods, Supplementary Table 1 and ref. 17) to infer the timing of extinction for 28 identified megafauna species (plus 8 indeterminate species identified only to genus) grouped into 16 genera (other genera do not present enough high-quality-rated fossil ages to compute the extinction window; Table 1) and first human occurrence. We applied these models to 659 megafauna fossil records and 438 archaeological records extracted from the new FosSahul database (see author information) by first aggregating species into genera and then computing the extinction window for each genus. Each method returns an extinction window (temporal confidence interval) for each taxon; thus, we calculated a window of cross-model agreement through time (that is, for every year from 120 to 0 kyr, we calculated how many models predicted extinction for a given taxon) under the assumption that higher cross-model agreement decreases uncertainties in extinction-window estimates^43^. From the 2,138 records of extinct fauna species in the database, each fossil was quality rated following an A* to C scale (from ‘high quality' to ‘unreliable') based on objective criteria^20^, including reliability in sample pretreatment and measurement (see details below). We used only A* and A quality-rated data and further excluded all data for which ages were obtained from materials in depositional context below or above the fossil(s) of interest. We used the same methodology to extract data from the 6,349 archaeological records. There are few human skeletal remains in the database; thus, human presence is largely restricted to artefacts related to human activities (for example, hearth charcoal, shell middens, stone tools and rock art). We excluded an age estimate of 62±6 kyr from Lake Mungo, because it is highly contested^44^,^45^. Including this age would push back our first human appearance estimate to about 62 kyr (see Supplementary Fig. 3), but does not affect our conclusion that megafauna extinctions are not correlated with climate variation. Its inclusion would result in a longer period of human–megafauna coexistence (18.5 kyr), thus extending the timeframe by about 5 kyr for humans to extirpate megafauna species continent wide. Similarly, the youngest *Genyornis* ages should be treated with caution, because ^14^C ages on carbonate materials are sensitive to exchange with younger carbon; such ages are potentially ‘minimum' ages and the true ages could be older than the estimated peak of extinction, but this complication does not affect our conclusions. We calibrated all ^14^C dates to calendar years before present using the Southern Hemisphere Calibration curve (ShCal13) and Marine Calibration curve (Marine13 for marine shells) from the OxCal radiocarbon calibration tool Version 4.2 (see https://c14.arch.ox.ac.uk).

### Quality rating of fossil ages

The quality-rating scheme is based on a two-stage set of objective criteria (see full method description and justification in ref. 20). An initial assessment is made of the reliability of an age, based on the dating procedure used, followed by an evaluation of the confidence in the stratigraphic relationship (referred to as ‘association') of the dated material to the target megafaunal taxon or archaeological event.

The ages obtained using five commonly used geochronology techniques in Sahul were rated for their quality^20^, with reliable ages coded as m* or m.

*Radiocarbon dating*. Reliable ages can be obtained by applying ultrafiltration, ninhydrin or XAD-2 protocols to individual amino acids or well-preserved bone and dentin collagen, and strong oxidation reagents (acid chlorate or dichromate) to charcoal. Reliable ages can also be obtained from cellulose isolated from wood, seeds, macrofossils and gut contents, and from the carbonate fractions of shells and corals with insignificant recrystallization, as assessed by X-ray diffraction.

*Amino acid racemization dating*. Reliable ages can be obtained from eggshells and otoliths of the target species, provided they have acted as chemically closed systems (i.e., no exchange in amino acids following burial of remains). Multiple analyses should be reproducible with low uncertainties and age calibration requires the availability of independent (and reliable) age estimates.

*Uranium-series dating*. Reliable ages can be obtained from materials that behave as chemically closed systems or as open systems when combined with modelling of uranium-migration processes. Reliable ages can be obtained from fossil teeth using coupled uranium-series/electron-spin resonance dating.

*Electron-spin resonance dating*. Reliable ages for tooth enamel can be obtained when combined with uranium-series modelling or if the internal dose rate due to uranium in dentine and enamel is <10% of the total dose rate.

*Luminescence dating*. Reliable ages can be obtained using the optically stimulated luminescence signal from individual grains and multi-grain aliquots of quartz or feldspar if the sediments were well bleached by sunlight before deposition. Reliable single-grain ages can also be obtained for partially bleached sediments using appropriate models.

The second step of the quality-rating scheme attributes a final A*/A rating to each m*/m age ranked in the first step (that is, to ages that satisfy the high-quality criteria for sample pretreatment and dating), based on the strength of association of the dated material to the target species of megafauna or the archaeological remains. Direct dating of megafaunal fossils can give A* or A ages, provided they satisfy the above criteria, as association is assured. However, indirect ages require careful checking for the strength of association. Such ages can be reliable if samples are recovered from contexts with high stratigraphic integrity and where fossils or other dated materials show no evidence of reworking (for example, burial sediments containing the articulated skeleton of a target species).

### Climate variables

The EPICA ‘Dome C' record provides an index of glacial/interglacial temperature fluctuations in Antarctica and has been used previously to explore potential drivers of megafauna extinctions in Sahul^7^. The EPICA initiative has provided the longest ice-core climate record yet, by drilling through 3,270-m-thick ice at a site known as ‘Dome C' in central East Antarctica^22^. As the time resolution of the core increases towards the present fluctuations on short time scales are more visible in recent parts of the record and can lead to misinterpretation of climate variability if the sampling bias is not corrected, we therefore resampled at an interval of 3 kyrs (to ensure at least 5 temperature values were available to calculate mean deviation per window) over 1,000 iterations and we calculated the mean temperature deviation for each interval and iteration.

The climate of a large part of eastern and northern Sahul is sensitive to changes in ENSO activity^46^; thus, metrics describing variation in the strength of ENSO events (such as ENSOp) are a good proxy for shifts in precipitation and temperature, and for changes in aridity. We extracted modelled ENSOp variation directly from Tudhope *et al*.^23^, who estimated ENSOp variability from application of the Zebiak–Cane coupled ocean–atmosphere model forced only by changing orbital parameters. ENSOp is an extracted sea surface temperature signal from the Nino 3 region, for which the higher power inference means more important effects of El Niño events (that is, increase in rainfall across the east-central and eastern Pacific and drier-than-normal conditions over northern Australia, Indonesia and the Philippines).

We also included temperature and precipitation proxies based on a hindcast of the HadCM3 global circulation model focussed specifically on Sahul. This model consists of linked atmospheric, ocean and sea ice models at a spatial resolution of 2.5° latitude × 3.75° longitude resampled at a 1 × 1° resolution^47^. The temporal resolution of the raw data is 1 kyr slices back to 22 kyr, 2 kyr from 22 to 80 kyr and 4 kyr to 120 kyr; thus, we ran our analyses using a 4-kyr running mean (Fig. 1e, f and Supplementary Fig. 1). We tested whether both the EPICA record and ENSOp data are representative of Sahul's palaeoclimate by calculating the spatial correlation of HadCM3 model outputs (temperature and precipitation) over the last 120 kyr for each gridcell to both EPICA and ENSOp. The strong correlation between these variables justifies the use of both EPICA and ENSOp as appropriate measures of climate variation in Sahul (Supplementary Figs 5 and 6). We also used the HadCM3 palaeoclimate simulation data to calculate vectors of climate velocity (km per year) for mean annual temperature (°C) and mean annual precipitation (mm per day) following established methods^34^. Climate velocity describes the rate (and direction) that an organism would need to migrate to maintain an isocline of a temperature and precipitation variable^34^. This proxy is often considered synonymous with the rate of climate displacement for a species, such that the higher the velocity, the less probable a species will keep pace with its shifting climatic niche and thus survive. Climate velocity is considered to be more biologically relevant than the use of climate anomalies, because it accounts for regional changes in climate and the ability of topographic heterogeneity to buffer biota against these changes^34^. We divided the rate of climate change through time (°C per year) by the spatial gradient in climate at that location (°C km^−1^).

### Information-theoretic ER

We fitted two regression models to the estimated number of extinct genera as a function of climate variables (described by our six proxies; Fig. 1d–h) at the same date. The first model includes a slope parameter that assumes a linear relationship between the number of genera going extinct and the climate conditions at a time step of 1,000 years. The second model is a simpler, mean-field approach, with only an intercept that assumes no climate–extinction relationship (the ‘null' model). The ER provides a way of explicitly evaluating the parameter bias-corrected likelihood of the null hypothesis (that is, no correlation between the number of extinct genera and climate variation) against the alternative (i.e., correlation between climate variation and the number of extinct genera). The ER is calculated as the sample size-adjusted AIC_*c*_ weight of the slope model divided by AIC_*c*_ weight of the competing model (in our case, the intercept-only model). Higher ERs (>3, see the ER interpretation scale in ref. 27) provide stronger support for the alternative hypothesis (model with slope) versus the null hypothesis, meaning that more genera went extinct as climate variation increased; for example, a strongly correlated climate variable might yield an ER>150. We also accounted for a possible temporal lag between climate conditions and megafauna extinctions, and for uncertainties in climate variables. We calculated the temporal lag by regressing against climate from 0 to 20,000 years (at a 2 kyr time step=11 temporal lag scenarios) for the period earlier than 35 kyr ago, which has the maximum number of extinct genera (Fig. 1a). For each of these scenarios, we generated 1,000 new sets of climate values resulting in 1,000 random resamples of the climate data within their confidence intervals (11 scenarios of ‘temporal lag' × 1,000 random resamples of each climate-lag data set within their confidence intervals=11,000 sets of climate values for each climate proxy). We refitted both linear models to the estimated number of genera going extinct as a function of these new sets of climate values and recalculated their ERs.

**Data availability:** Models and data used in analyses are available on request to F.S. (frederik.saltre@adelaide.edu.au) from the Global Ecology Laboratory, School of Biological Sciences at the University of Adelaide. Sahul's megafauna data set is also available online in the AEKOS data repository (DOI:10.4227/05/564E6209C4FE8).

## Additional information

**How to cite this article:** Saltré, F. *et al*. Climate change not to blame for late Quaternary megafauna extinctions in Australia. *Nat. Commun.* 7:10511 doi: 10.1038/ncomms10511 (2016).

## Supplementary Material

Supplementary Information Supplementary Figures 1-6, Supplementary Table 1, Supplementary Methods and Supplementary References

## Figures and Tables

**Table 1:** Fossil records and archaeological evidence extracted from the FosSahul database used to infer ages for megafauna extinction and first human occurrence.

**Genera**	**Species** ***(n*** **records/species)**	**Total records**	**Total time range (kyr±1*****σ*****)**
*Congruus*	*C. kitchenerii* (5)	5	63±9 – 136.8±2.6
*Diprotodon*	Indet. (20), *D. optatum* (9)	29	44.2±5.7 – 1018.5±122.5
*Genyornis*	*G. newtoni* (252)	252	35±0.4 – 139.9±0
*Macropus*	Indet. (15), *M. ferragus* (3), *M. nov* (8), *M. pearsoni* (1)	27	43.3±0.5 – 876±206
*Megalibgwilia*	Indet. (1), *M. ramsayi* (6)	7	56±4 – 136.8±2.6
*Metasthenurus*	*M. newtonae* (8)	12	56±4 – 535±49
*Palorchestes*	Indet. (1), *P. azael* (4)	5	56±4 – 257±21
*Phascolonus*	*P. gigas* (6)	8	46±6 – 122±22
*Procoptodon*	Indet. (1), *P. browneorum* (28), *P. gilli* (16), *P. goliah* (12)	57	40±0.2 – 535±49
*Protemnodon*	Indet. (6), *P. anak* (16), *P. brehus* (19), *P. roechus* (13)	54	40.8±0.3 – 535±49
*Sarcophilus*	*S. laniarius* (9)	12	46±6 – 535±49
*Simosthenurus*	Indet. (2), *S. baileyi* (1), *S. maddocki* (7), *S. occidentalis* (26), *S. pales* (11)	47	44.9±1.3 – 535±49
*Sthenurus*	Indet. (2), *S. andersoni* (13), *S. atlas* (2), *S. stirlingi* (1), *S. tindalei* (3)	21	52±8 – 535±49
*Thylacinus*	*T. cynocephalus* (35)	35	3.5±0.1 – 297±9
*Thylacoleo*	*T. carnifex* (37)	37	44.9±1.3 – 535±49
*Zygomaturus*	*Z. trilobus* (34)	34	44.9±1.3 – 535±49
*Homo*	*H. sapiens* (436)	436	0.07±0.04 – 55.5±8.2

Indet, indeterminate species identification.

Age reliability was assessed following established quality-rating criteria^20^ (see details in Methods). Genera with at least four reliably dated fossil records (the minimum number required to run ensemble-hindcasting models^17^) were used for extinction-window estimates (Fig. 1a – c); this resulted in the removal of seven genera (*Borungaboodie*, *Progura*, *Propleopus*, *Troposodon*, *Varanus*, *Wonambi* and *Zaglossus*) from our analyses.
