# Supplementary material for: Climate change not to blame for late Quaternary megafauna extinctions in Australia
Source: Nat Commun. 2016 Jan 29;7:10511. doi: 10.1038/ncomms10511 (PMC4740174; doi:10.1038/ncomms10511)
Supplement: Supplementary Information — Supplementary Figures 1-6, Supplementary Table 1, Supplementary Methods and Supplementary References [file ncomms10511-s1.pdf]

Supplementary Figures

Supplementary Figure 1 – Boxplot of information-theoretic evidence ratios (*ER*) comparing two regression models (the first with a slope parameter and the second with only an intercept) fitted to variation in six climate proxies against the number of extinct species (estimated from the distribution of the last fossil for each taxon) between 35 and 120 kyr ago (kyr = 10^3 years) as a function of a temporal lag between the date of species extinction and climate variation. The model with the slope parameter assumes a linear positive relationship between the number of extinct species over time and climate conditions, whereas the simpler model with only an intercept assumes no relationship. The *ER* is calculated as the sample size-adjusted Akaike's information criterion [AIC_c] weight of the slope model divided by the AIC_c weight of the intercept-only model. A higher *ER* (>> 3, see the evidence ratio interpretation scale in ref 1) indicates more evidence for the slope versus the intercept-only model, indicating that as climate variation increased, more taxa went extinct. The temporal lag is calculated by regressing the number of species going extinct against climate from 0 to 20,000 year (at a 2-kyr time step) earlier than 35 kyr (i.e., the period showing the maximum number of extinct taxa; Fig. 1a). For each temporal lag scenario, we accounted for climate uncertainty by re-fitting both linear models using 1,000 new sets of climate values resulting in 1,000 random resamples of the climate data within their confidence intervals (per 1-kyr window).

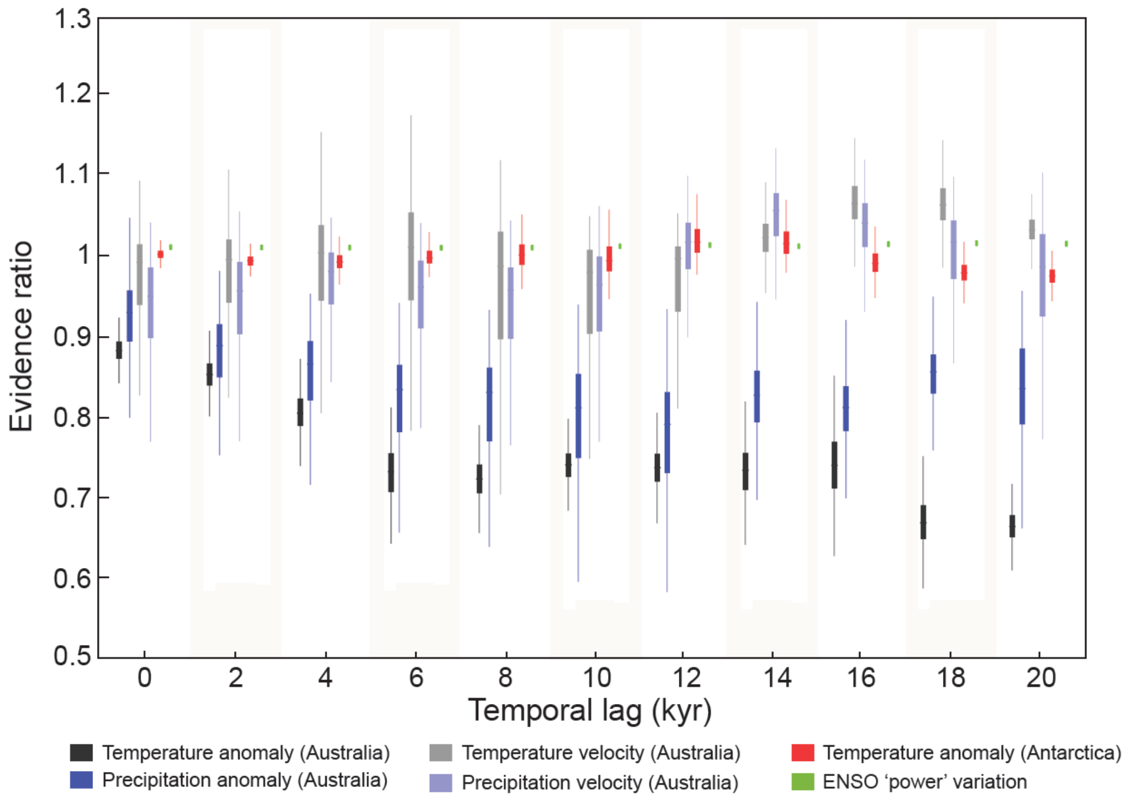

Supplementary Figure 2 – Geographic distribution of dated (a) megafauna and (b) archaeological sites related to human activities in Sahul (i.e., Australia and New Guinea). We used only high quality-rated dated records of both human and megafauna taxa in this study (see *Methods* in main text)

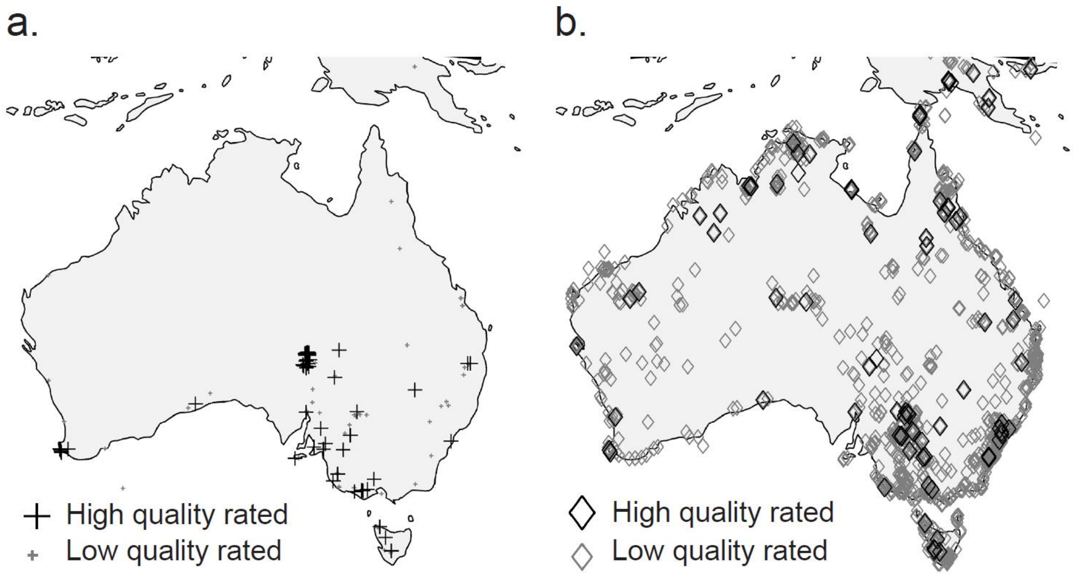

Supplementary Figure 3 – Period of human-megafauna overlap in Sahul accounting for the highly debated date of 62 ± 6 kyr (kyr = 10^3 years) from Lake Mungo[2, 3, 4]. Distribution of extinction dates for all megafauna taxa with a model agreement > 50% (grey barplot, left *y*-axis) and distribution of the percentage of model agreement to infer the date of first human occurrence (blue barplot, right *y*-axis). Marine Isotope Stages (MIS)[5] 1 to 5 are shown across the top axis for temporal reference.

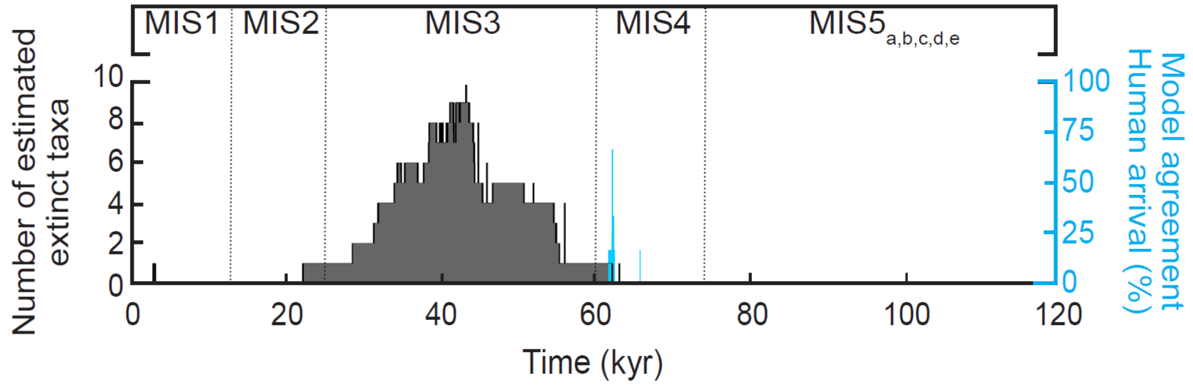

Supplementary Figure 4 – Isotope δ^{18}O values (100-year averaged data) extracted from the WAIS Divide Core 06A[6] over the last 68 kyr (kyr = 10^3 years). Although we are aware of this recent work, we have not used this record to test correlation between the climate variation and the estimated number of extinct species through time because of the redundancy with the EPICA Dome C ice core in central-east Antarctica[7], which is a longer record and has the same trends for the period of overlap. Marine Isotope Stages (MIS)[5] 1 to 5 are shown across the top axis for temporal reference.

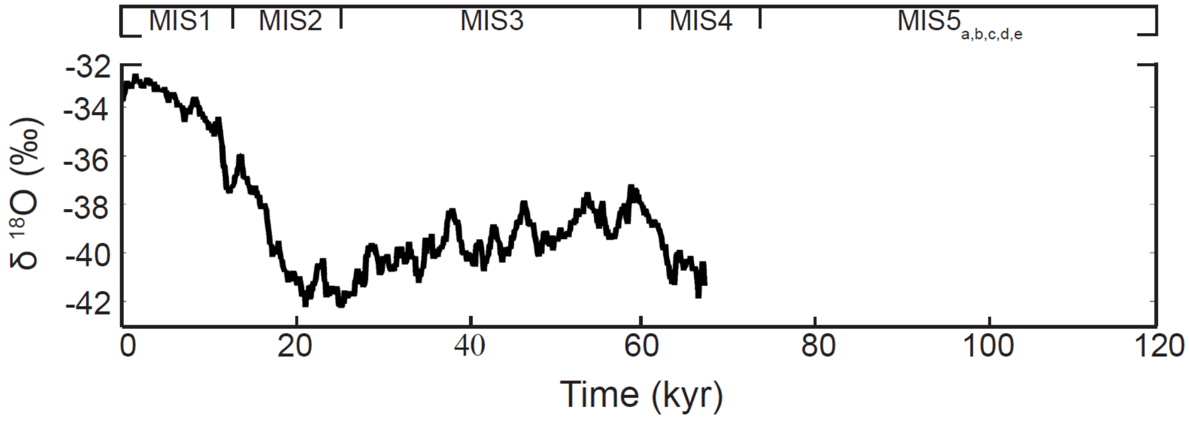

Supplementary Figure 5 – Maps of correlation between climate in Sahul at a 1 × 1 ° spatial resolution and glacial/interglacial temperature fluctuations in Antarctica (Fig. 1 in the main text) over the last 120 kyr (kyr = 10^3 years, a and b), Marine Isotope Stage 3 (c and d) and 5 (e and f)[5]. The correlation is calculated using the Spearman's rank correlation coefficient applied to mean annual temperature (a, c and e) and precipitation (b, d and f) anomalies relative to the present day at each gridcell, calculated from HadCM3 palaeoclimate simulations[8], and temperature fluctuations in Antarctica are calculated from the Antarctica EPICA Dome C ice core[7] and corrected for time-resolution sampling bias.

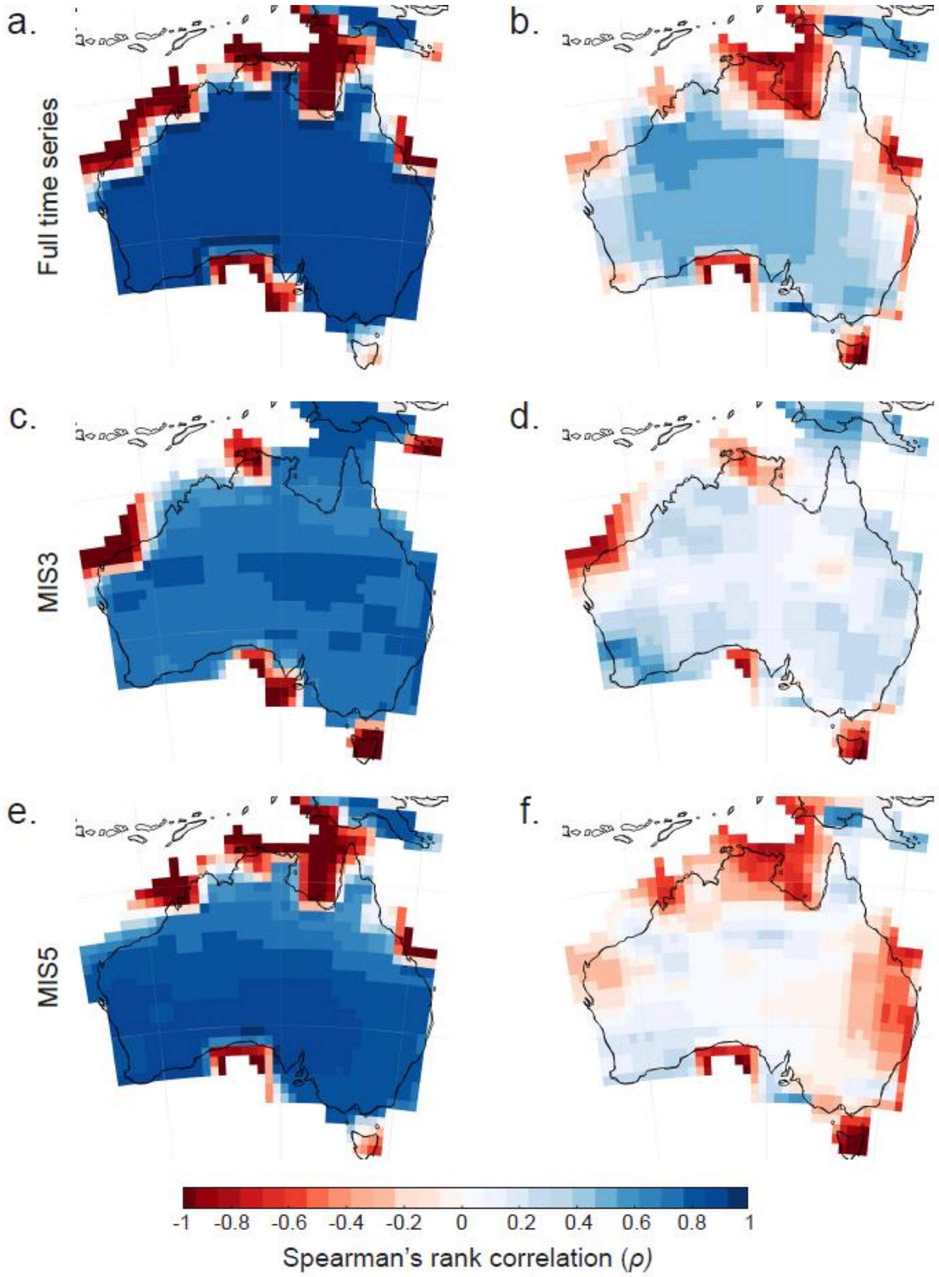

Supplementary Figure 6 – Maps of correlation between climate in Sahul at a 1 × 1 ° spatial resolution and variation in El Niño/Southern Oscillation power (ENSOp, see Fig. 1 in the main text) over the last 120 kyr (kyr = 10^3 years, a and b), Marine Isotope Stage 3 (c and d) and 5 (e and f)[5]. The correlation is calculated using the Spearman's rank correlation coefficient applied to mean annual temperature (a, c and e) and precipitation (b, d and f) anomalies relative to the present day at each gridcell, calculated from HadCM3 palaeoclimate simulations[8], and variation in ENSOp is estimated from the Zebiak-Cane coupled ocean-atmosphere model forced only by changing parameters[9].

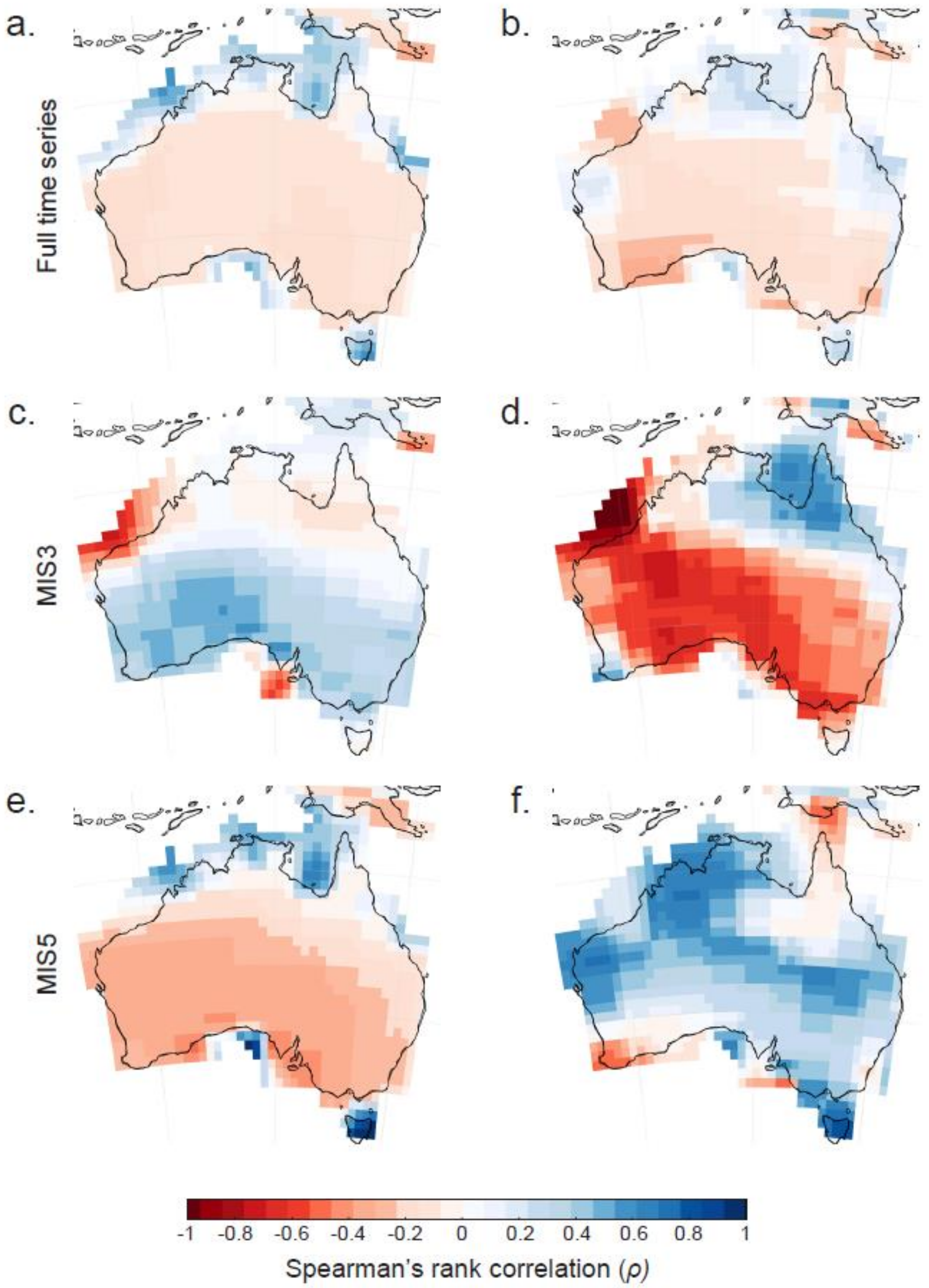

Supplementary Tables

Supplementary Table 1 – Inferential methods used to estimate the extinction timing of sixteen megafauna genera and the time of first human occurrence in Sahul – see Figure 1 in main text. Each variable is explained in detail in the Supplementary Method 1.

**Method**	**Reference**	**Formula**
Strauss & Sadler	Strauss & Sadler[10]	$T_{ext} = T_n + \lambda \times (T_n - T_H)$ $\lambda = (1-\alpha)^{-1/(H-1)} - 1$
McCarthy	McCarthy[11]	$\sum_{i=1}^{i=T_{ext}} e_i = \frac{\sum_{i=1}^{i=T_n} e_i}{\alpha^{(1/H)}}$
Marshall	Marshall[12]	$\sum_{i=T_n}^{i=T_{ext}} e_i = \lambda \sum_{i=1}^{i=T_n} e_i$
Solow	Solow *et al.*[13]	$T_{ext(min,max)} = argmax \prod_{i \leq H} \frac{\Phi\left({(T_i - T_{\mathrm{ext(min)}})}/{\sigma_T} \right) - \Phi\left({(X_i - T_{\mathrm{ext(max)}})}/{\sigma_T} \right)}{T_{\mathrm{ext(max)}} - T_{\mathrm{ext(min)}}}$
McInerny	McInerny *et al.*[14]	$T_{ext} = T_n + \log_{[1-(H/T_n)]}(1-\alpha)$
GRIWM	Bradshaw *et al.*[15]	$T_{ext} = \frac{1}{L} \sum_{l=1}^{L} \sum_{i=1}^{H-1} \omega_{i(l)} \frac{\log \alpha}{\log\left(1 - \frac{H}{T_n}\right)} + (T_{l,n})$

Supplementary Methods

Detail of models used in the ensemble-hindcasting approach

Except for GRIWM (Gaussian-Resampled Inverse-Weighted McInerny) and Solow's model described in Saltré *et al.*[16], all methods (see supplementary table hereafter) were adapted to the fossil time series and dated artefacts related to human activities from Rivadeneira *et al*[17]. T_{ext} is the estimated extinction time, $T_1, .., T_n$ are n fossil dates of presence sorted such that T_n is the date of the youngest record from the fossil time series, H is the total number of dated records in the time series, and α is the confidence level (median estimates from α = 0.05 and their confidence interval ranging from α = 0.975 and α = 0.025). Solow's model estimates a confidence interval defined as $T_{ext_{(min,max)}}$ encompassing T_{ext}. Φ is the cumulative distribution function of the standard normal distribution and σ_T represents the dating error — assumed to be constant along the fossil time series. However, Solow's equation (like Strauss and Sadler's and McInerny's) assumes a uniform probability of record occurrence over time, an assumption that might often be violated because the extinction vortex[18] acts to reduce the probability of discovering fossil records near the terminal date[16]. McCarthy's and Marshall's methods account for this effect using e_i, the sampling effort (i.e., a function of sampling probability that depends on the time series used to calculate extinction time[16, 17]) in the ith year. Instead of calculating this sampling effort, GRIWM hypothesizes that the most-recent records would be more influential on the sighting rate as extinction is approached so that $\omega_i = \frac{1}{T_n - T_i} \Big/ \sum_{j=1}^{H-1} \frac{1}{\frac{1}{T_{l,n} - T_{l,i}}}$, where ω_i (*i*) gives more 'weight' to the most recent dates by inversely weighting the contribution of each dated record to T_{ext} given its temporal distance from the most recent record and (*ii*) resamples '*L*' times (arbitrarily set at 10,000) each radiometric date in the series from a Gaussian distribution (with mean = $T_1, .., T_n$ and standard deviation =$\sigma_1, .., \sigma_n$) assuming normally distributed errors.

Supplementary References

1. Kass RE, Raftery AE. Bayes Factors. *J. Am. Stat. Assoc.* **90**, 773-795 (1995).
2. Bowler JM, Magee JW. Redating Australia's oldest human remains: a sceptic's view. *J. Hum. Evol.* **38**, 719-726 (2000).
3. Gillespie R, Roberts RG. On the reliability of age estimates for human remains at Lake Mungo. *J. Hum. Evol.* **38**, 727-732 (2000).
4. Thorne A*, et al.* Australia's oldest human remains: age of the Lake Mungo 3 skeleton. *J. Hum. Evol.* **36**, 591-612 (1999).
5. Thompson WG, Goldstein SL. A radiometric calibration of the SPECMAP timescale. *Quat. Sci. Rev.* **25**, 3207-3215 (2006).
6. Wais Divide Project Members. Precise interpolar phasing of abrupt climate change during the last ice age. *Nature* **520**, 661-665 (2015).
7. EPICA community members. Eight glacial cycles from an Antarctic ice core. *Nature* **429**, 623-628 (2004).
8. Singarayer JS, Valdes PJ. High-latitude climate sensitivity to ice-sheet forcing over the last 120kyr. *Quat. Sci. Rev.* **29**, 43-55 (2010).
9. Tudhope AW*, et al.* Variability in the El Niño-Southern Oscillation Through a Glacial-Interglacial Cycle. *Science* **291**, 1511-1517 (2001).
10. Strauss D, Sadler P. Classical confidence intervals and Bayesian probability estimates for ends of local taxon ranges. *Math. Geol.* **21**, 411-427 (1989).
11. McCarthy MA. Identifying declining and threatened species with museum data. *Biol. Conserv.* **83**, 9-17 (1998).
12. Marshall CR. Confidence intervals on stratigraphic ranges with nonrandom distributions of fossil horizons. *Paleobiology* **23**, 165-173 (1997).
13. Solow AR, Roberts DL, Robbirt KM. On the Pleistocene extinctions of Alaskan mammoths and horses. *Proc. Natl. Acad. Sci. U.S.A.* **103**, 7351-7353 (2006).
14. McInerny GJ, Roberts DL, Davy AJ, Cribb PJ. Significance of sighting rate in inferring extinction and threat. *Conserv. Biol.* **20**, 562-567 (2006).
15. Bradshaw CJA, Cooper A, Turney CSM, Brook BW. Robust estimates of extinction time in the geological record. *Quat. Sci. Rev.* **33**, 14-19 (2012).
16. Saltré F*, et al.* Uncertainties in specimen dates constrain the choice of statistical method to infer extinction time. *Quat. Sci. Rev.* **112**, 128-137 (2015).
17. Rivadeneira MM, Hunt G, Roy K. The use of sighting records to infer species extinctions: an evaluation of different methods. *Ecology* **90**, 1291-1300 (2009).
18. Fagan WF, Holmes EE. Quantifying the extinction vortex. *Ecol. Lett.* **9**, 51-60 (2006).
